# Low-Molecular-Weight Heparin Plus Insulin in Hypertriglyceridemic Acute Pancreatitis: A Randomized Clinical Trial

**DOI:** 10.1001/jamanetworkopen.2025.42124

**Published:** 2025-11-07

**Authors:** Wenhua He, Ling Ding, Zhijun Liu, Ming’e Hua, Yuming Zhou, Min Gong, Jinyi Sheng, Ximing Wu, Huizhen Fan, Hongchun Shu, Ruilan Wang, Linting Xun, Caibin Huang, Feng Guo, Chunshui Cao, Zuan Zhan, Huifang Chen, Xing Qian, Qinghong Liu, Qiang Li, Zhihua Tang, Yanyan Xiong, Ping Xie, Bingjun Yu, Jianhua Wan, Huajing Ke, Yao Wu, Yang Hu, Jiaoyu Ai, Xin Huang, Yupeng Lei, Huifang Xiong, Zhijian Liu, Yong Zhu, Lingyu Luo, Liang Xia, Lu Ke, Wenhao Cai, Wei Huang, Weiqin Li, John Windsor, Robert Sutton, Nonghua Lv, Yin Zhu

**Affiliations:** 1 Department of Gastroenterology, First Affiliated Hospital, Jiangxi Medical College, Nanchang University, Nanchang, China; 2 Department of Gastroenterology, Xinyu People’s Hospital, Xinyu, China; 3 Department of Gastroenterology, Fuzhou First People’s Hospital, Fuzhou, China; 4 Department of Emergency, Ganzhou People’s Hospital, Ganzhou, China; 5 Department of Gastroenterology, Pingxiang People’s Hospital, Pingxiang, China; 6 Department of Gastroenterology, Jingdezhen First People’s Hospital, Jingdezhen, China; 7 Department of Gastroenterology, Yingtan People’s Hospital, Yingtan, China; 8 Department of Gastroenterology, Yichun People’s Hospital, Yichun, China; 9 Department of Gastroenterology, Shangrao People’s Hospital, Shangrao, China; 10 Department of Gastroenterology, First Affiliated Hospital of Shanghai Jiaotong University, Shanghai, China; 11 Department of Gastroenterology, First People’s Hospital of Yunnan, Kunming, China; 12 Department of Gastroenterology, First Affiliated Hospital of Gannan Medical College, Ganzhou, China; 13 Intensive Care Unit, Sir Run Run Shaw Hospital, Hangzhou, China; 14 Department of Emergency, First Affiliated Hospital of Nanchang University, Nanchang, China; 15 Center of Severe Acute Pancreatitis (CSAP), Department of Critical Care Medicine, Nanjing Jinling Hospital, Affiliated Hospital of Medical School, Nanjing University, Nanjing, China; 16 West China Centre of Excellence for Pancreatitis, Institute of Integrated Traditional Chinese and Western Medicine, West China-Liverpool Biomedical Research Centre, West China Hospital, Sichuan University, Chengdu, China; 17 Surgical and Translational Research Centre, Faculty of Medical and Health Sciences, University of Auckland, Auckland, New Zealand; 18 Liverpool Pancreatitis Research Group, Institute of Systems, Molecular and Integrative Biology, University of Liverpool and Liverpool University Hospitals National Health Service Foundation Trust, Liverpool, Merseyside, UK

## Abstract

**Importance:**

Although insulin is widely used for early lipid lowering in hypertriglyceridemic acute pancreatitis, the clinical benefit of heparin combined with insulin remains unclear.

**Objective:**

To examine whether low-molecular-weight heparin (LMWH) combined with insulin is superior to insulin alone in improving clinical outcomes in hypertriglyceridemic acute pancreatitis.

**Design, Setting, and Participants:**

This multicenter, open-label, parallel-group, superiority randomized clinical trial was conducted from September 21, 2019, to April 2, 2023, across 13 tertiary hospitals in China. The duration of follow-up was 30 days after randomization. Patients aged 18 to 85 years were eligible if they met the diagnostic criteria for acute pancreatitis, presented with baseline serum triglyceride levels between 1000 and 3550 mg/dL, and were admitted within 48 hours of symptom onset.

**Interventions:**

Participants received either subcutaneous LMWH (4000 IU every 12 hours for 3 days) plus insulin (LMWH plus insulin group) or insulin alone (insulin group).

**Main Outcomes and Measures:**

The primary end point was a composite of new-onset organ failure (defined as organ failure occurring after randomization but absent during the 24-hour prerandomization period) and/or all-cause mortality within 30 days after randomization.

**Results:**

Among 533 randomized patients (median [IQR] age, 39 [33-46] years; 406 [76.2%] male), 264 were randomized to LMWH plus insulin and 269 to insulin alone. The primary end point occurred in 66 (25.0%) of the LMWH and insulin group vs 76 (28.3%) of the insulin group (relative risk [RR], 0.89; 95% CI, 0.67-1.17; *P* = .40). Both groups achieved the triglyceride target (<500 mg/dL) in a median (IQR) of 2 (1-3) days (*P* = .94). Safety outcomes were similar between groups, including new-onset bleeding events (RR, 0.61; 95% CI, 0.15-2.53; *P* = .73), triglyceride rebound (RR, 0.53; 95% CI, 0.28-1.01; *P* = .05), and drug-related adverse events (RR, 0.79; 95% CI, 0.30-2.10; *P* = .64).

**Conclusions and Relevance:**

In this randomized clinical trial, the combination of LMWH and insulin failed to demonstrate superior efficacy vs insulin alone in patients with hypertriglyceridemic acute pancreatitis. LMWH might not be necessary for early lipid-lowering in hypertriglyceridemic acute pancreatitis.

**Trial Registration:**

ChiCTR.org.cn Identifier: ChiCTR1900023640

## Key Points

**Question:**

Is low-molecular-weight heparin combined with insulin superior to insulin alone in improving clinical outcomes in hypertriglyceridemic pancreatitis?

**Findings:**

In this multicenter randomized clinical trial of 533 adults with acute pancreatitis, low-molecular-weight heparin with insulin was not superior to insulin alone in reducing new-onset organ failure and/or mortality.

**Meaning:**

Low-molecular-weight heparin might not be necessary for early lipid-lowering in hypertriglyceridemic acute pancreatitis.

## Teaser

This randomized clinical trial of adults with acute pancreatitis assesses whether low-molecular-weight heparin combined with insulin is superior to insulin alone in reducing new-onset organ failure and/or mortality.

## Introduction

Acute pancreatitis (AP) is a common gastrointestinal disease with diverse origins, including gallstones, alcohol consumption, and hypertriglyceridemia (HTG).^1^ Globally, HTG represents the third most common cause of AP, accounting for approximately 4.7% of cases, with an increasing incidence.^2,3^ In China, HTG has become the second leading cause, now responsible for more than 25% of AP cases.^4,5^ Evidence suggests that, in contrast to other origins, hypertriglyceridemic acute pancreatitis (HTG-AP) appears to be more severe, manifesting as a higher incidence of systemic inflammatory response syndrome (SIRS),^6^ persistent organ dysfunction,^7^ local complications, and increased mortality.^8^

Given these clinical consequences, optimal triglyceride-lowering strategies, including insulin, heparin, heparin combined with insulin, and blood purification, remain a subject of active investigation.^9^ Current guidelines provide no specific recommendations for early triglyceride-lowering therapies due to insufficient high-quality evidence.^10,11,12,13^ Although insulin is widely used for early lipid-lowering in HTG-AP, the clinical benefit of heparin combined with insulin remains controversial. Concerns persist regarding heparin’s prolonged use, including potential rebound elevation of triglyceride (attributable to endothelial lipoprotein lipase depletion)^14^ and increased pancreatic hemorrhage risk.^15^ However, the bleeding risk of low-molecular-weight heparin (LMWH) was significantly lower than that of unfractionated heparin,^16^ and it exerts anti-inflammatory effects.^17^

A multicenter, prospective study^18^ found that LMWH can reduce the incidence of complications, mortality, and hospital stay in severe AP, and no hemorrhagic complications occurred. A prior randomized clinical trial^19^ found that LMWH combined with insulin achieved target plasma triglyceride levels (<500 mg/dL [to convert to millimoles per liter, multiply by 0.0113]) in approximately 48 hours in patients with HTG-AP, with reduced respiratory failure incidence compared with hemofiltration and without a serious rebound phenomenon and severe adverse effects. This multicenter randomized clinical trial assessed whether LMWH combined with insulin is superior to that of insulin alone in reducing new-onset organ failure and/or mortality in patients with HTG-AP.

## Methods

### Study Design

This investigator-initiated, multicenter, open-label, parallel-group, superiority randomized clinical trial was conducted across 13 tertiary hospitals in China (eTable 1 in Supplement 1). The trial was approved by the Medical Ethics Committee of the First Affiliated Hospital of Nanchang University and all participating sites before patient enrollment and then was registered at the Chinese Clinical Trial Registry on June 5, 2019. The trial adhered to the Declaration of Helsinki^20^ and Chinese law regarding research involving humans. All participants or their legal representatives provided written informed consent. The trial protocol is available in Supplement 2. This trial conformed to the Consolidated Standards of Reporting Trials (CONSORT) reporting guidelines.^21^

### Study Participants

From September 21, 2019, to April 2, 2023, a total of 941 patients were assessed for eligibility. Patients aged 18 to 85 years were eligible if they met the diagnostic criteria for AP according to the 2012 Revised Atlanta Classification,^22^ presented with baseline serum triglyceride levels between 1000 and 3550 mg/dL, and were admitted within 48 hours of symptom onset. Exclusion criteria included other identified causes of AP (biliary or alcoholic), pregnancy or lactation, contraindications to LMWH, known hypersensitivity to heparin or insulin, use of any triglyceride-lowering drugs (such as fibrates or statins) between symptom onset and randomization, and requirement for blood purification due to acute kidney failure. We also excluded patients who required cardiopulmonary resuscitation for cardiac arrest, those with irreversible organ failure, and patients with serious chronic diseases. Data on race were not collected because the study was conducted within a single ethnic country (China), where reporting participants’ race or ethnicity is not standard practice for domestic research and was not required by our ethics approval.

### Randomization and Open-Label Design

We implemented a centralized randomization system for participant allocation. Eligible patients were randomly assigned in a 1:1 ratio to receive either LMWH plus insulin (LMWH plus insulin group) or insulin alone (insulin group). To maintain allocation concealment and minimize selection bias, an independent study coordinator at each site performed randomization and masked allocation assignments via a web-based central randomization software (Churun Information Technology Co Ltd). The researchers were responsible for screening, recruitment, dispensing medicine, and outcome assessment to the group allocation. In this open-label study, both participants and investigators were aware of treatment assignments. The statistical analyses were conducted by independent statisticians who were blinded to trial assignments.

### LMWH or Insulin Therapy

In the LMWH plus insulin group, participants received subcutaneous enoxaparin (4000 IU every 12 hours for 3 days).^14,19^ Insulin (Norheparin R, Novo Nordisk) therapy was administered as follows: for blood glucose level greater than 200 mg/dL (to convert to millimoles per liter, multiply by 0.0555), continuous intravenous infusion (2-6 U/h),^23,24^ monitor blood glucose closely, and target glucose levels of 140 to 180 mg/dL^25^; or for blood glucose levels of 200 mg/dL or less, intravenous insulin (6 U) with 5% dextrose (500 mL), twice a day for 5 days (if hypoglycemia occurs, change 5% dextrose to 10% dextrose). The insulin group received the insulin therapy as described for the LMWH plus insulin group.

### Concomitant Medications

When triglyceride levels reach less than 500 mg/dL (to convert to millimoles per liter, multiply by 0.0113) or at 5 days after enrollment, insulin will be stopped and oral lipid-lowering drugs will be administered in the 2 study groups. If the blood glucose level is still greater than 200 mg/dL, insulin will be maintained to control the blood glucose. Although LMWH administration was limited to 3 days (irrespective of triglyceride levels) in the LMWH plus insulin group, LMWH (4000 IU every 12 hours for 7-10 days) was administered as venous thromboembolism prophylaxis in patients at high thrombotic risk (Padua score ≥4) and with low bleeding risk in both groups.^17^ The dose that patients with LMWH received on each day are provided in eTable 2 in Supplement 1.

### Standard Treatment Regimen

Early treatments include fasting for 48 hours,^26^ fluid resuscitation, and analgesia.^10^ Patients were offered intensive care after organ failure. Antibiotics were given after clinically suspected or confirmed infection. For those demonstrating clinical tolerance (absence of severe abdominal pain, nausea, or vomiting), a low-fat soft diet was introduced. Patients with persistent intolerance to oral intake for 24 to 48 hours received enteral nutrition via nasogastric or nasojejunal tubes. Parenteral nutrition without fat emulsion was administered when enteral feeding was contraindicated, poorly tolerated, or insufficient to meet caloric needs. If the triglyceride levels were greater than 250 mg/dL^27^ at day 5 after randomization, oral lipid-lowering drugs (fibrates alone or in combination with statins or niacin) were administered. Patients with infected or symptomatic walled-off pancreatic necrosis were primarily managed with a step-up minimally invasive approach.

### End Points

The primary end point was a composite of new-onset organ failure (defined as organ failure occurring after randomization but absent during the 24-hour prerandomization period) and/or all-cause mortality within 30 days after randomization. Organ failure, including respiratory failure, circulatory failure, and kidney failure, was defined as a score of 2 or more using the modified Marshall scoring system.^22^ Secondary end points included time to achieve the triglyceride goal of less than 500 mg/dL, incidence of new-onset or persistent (lasting >48 hours) SIRS, complications, interventions, hospital stay, and costs. Safety outcomes included new-onset bleeding events, rebound elevation of serum triglyceride levels, and drug-related adverse events.

### Data Management

Prospective data collection was performed by the nominated investigators (1 or 2 in each participating center) using paper-based case report forms. Independent First Affiliated Hospital of Nanchang University investigators conducted biannual quality audits of all case report forms to verify data accuracy, completeness, and legibility. Identified discrepancies (missing, implausible, or inconsistent data) underwent investigator review and correction before database locking. We implemented a double-data entry system using EpiData software, version 3.1 (EpiData Association) with subsequent validation. The electronic database maintained deidentified participant information to ensure confidentiality. The First Affiliated Hospital of Nanchang University provided standardized training on data entry procedures and software use and assumed responsibility for data safety, privacy, and quality. An independent data and safety monitoring committee with no competing interests was responsible for overseeing the safety and quality of the data. The independent data and safety monitoring committee considered protocol adherence, participant withdrawal, and safety monitoring and made recommendations for continuation of the trial.

### Protocol Amendments

The initial version (2017.7) of the study protocol had a double-blind study design; after a multicenter discussion in November 2018, it was revised to single blind and published in *Pancreas*.^28^ Before study commencement, the protocol received approval from all participating institutional ethics committees. In May 2019, the trial design was modified from blinded to open-label due to impracticability in maintaining effective blinding. The sample size was increased from 476 to 540 following statistical advice. Due to the increased risk of bleeding in LMWH, acute kidney failure was added as an exclusion criterion by the Medical Ethics Committee on June 30, 2020. The revised protocol was approved by ethics committees and updated in the Chinese Clinical Trial Registry.

### Statistical Analysis

This study is designed as a superiority trial, and the sample size is calculated based on data from a previous clinical study.^19^ The occurrence rate of the new-onset organ failure and/or mortality of LMWH combined with insulin in patients with HTGP is 8.3%, whereas that for treatment with insulin alone in patients with HTGP is 17.9%. We estimate that a sample size of 490 participants will provide 80% power at a 2-sided α level of .05 to detect a 9.6% reduction in the composite primary end point. Given an attrition rate of 10%, we planned to randomize 540 patients in total (270 per group). The sample size was calculated using PASS software, version 11 (NCSS Software).

Baseline variables are expressed as medians (IQRs) or numbers (percentages). Primary analyses followed intention-to-treat principles and included all randomized participants. The per-protocol population excluded ineligible patients, protocol deviations, and consent withdrawals. The primary end point was analyzed using the χ^2^ test. The secondary end points and safety outcomes were evaluated with the χ^2^, Fisher exact, or Wilcoxon rank-sum tests as appropriate. Treatment effects are presented as relative risks (RRs) with 95% CIs. To evaluate the robustness of our findings, a post hoc sensitivity analysis of the primary end point was performed using robust Poisson regression adjusted for selected baseline covariates (sex; age; body mass index; duration from pain onset to admission; referral status; history of AP, hypertension, or diabetes; smoking or drinking status; agents used before AP onset; organ failures; triglyceride level; SIRS score; and Acute Physiology and Chronic Health Evaluation II score). Another post hoc sensitivity analysis of the primary end point was performed in patients who received LMWH for 3 days in the LMWH plus insulin group and those who received insulin alone in the control group using robust Poisson regression adjusted for selected baseline covariates. Post hoc subgroup analyses were performed based on age, baseline AP severity, baseline triglyceride level strata, and time from symptom onset to admission to explore heterogeneous treatment effects by testing interaction terms using logistic regression without adjustment for multiple testing. Missing data were reported descriptively (eTable 3 in Supplement 1), with no imputation performed. All statistical analyses were conducted using SPSS software, version 26.0 (SPSS Inc), with statistical significance set at a 2-sided α = .05.

## Results

### Baseline Characteristics

Among 533 randomized patients (median [IQR] age, 39 [33-46] years; 406 [76.2%] male), 264 randomized to LMWH plus insulin and 269 to insulin alone. The flowchart of the study is depicted in the Figure. Baseline characteristics were comparable between the 2 groups (Table 1).

**Figure.**
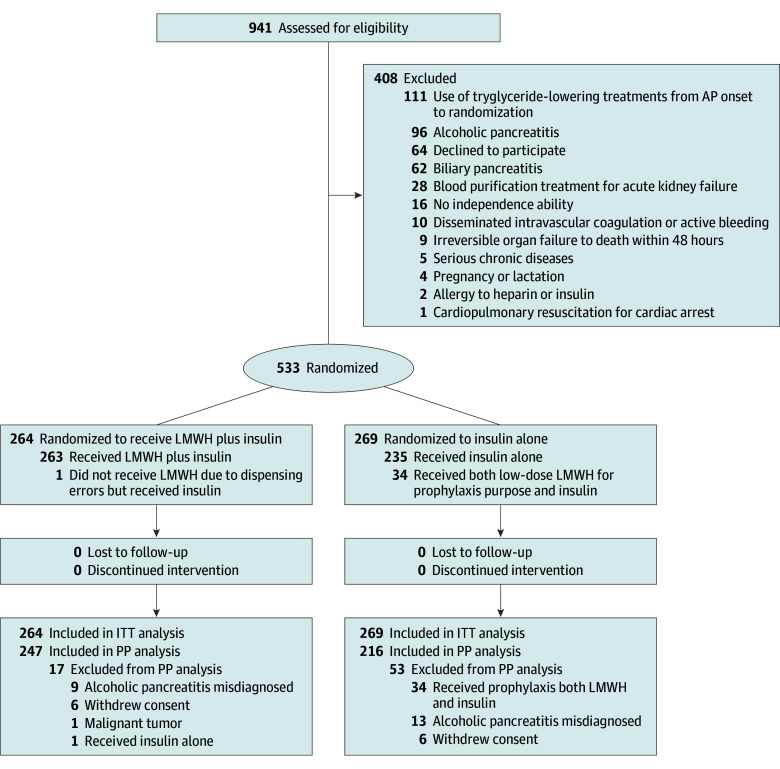
Study Flow Diagram AP indicates acute pancreatitis; ITT, intention to treat; LMWH, low-molecular-weight heparin; PP, per protocol.

**Table 1.** Baseline Characteristics of Patients in the Intention-to-Treat Population

Characteristic	Patients, No. (%)	
	LMWH plus insulin group (n = 264)	Insulin group (n = 269)
Sex		
Male	207 (78.4)	199 (74.0)
Female	57 (21.6)	70 (26.0)
Age, median (IQR), y	39 (33-46)	39 (33-46)
BMI, median (IQR)	26 (25-29)	26 (24-29)
Duration from pain onset to admission, median (IQR), h	17 (10-24)	16 (10-24)
Referral^a^	51 (19.3)	66 (24.5)
History of AP, median (IQR), No. of episodes	1 (0-2)	0 (0-2)
Hypertension	35 (13.3)	34 (12.6)
Diabetes	66 (25.0)	67 (24.9)
Smoker		
Never	182 (68.9)	183 (68.0)
Current	81 (30.7)	83 (30.9)
Past	1 (0.4)	3 (1.1)
Drinker^b^		
Never	175 (66.3)	184 (68.4)
Light	80 (30.3)	72 (26.8)
Heavy	9 (3.4)	13 (4.8)
Drugs use before AP onset		
Hypoglycemic agents	35 (13.3)	31 (11.5)
Hypotensive agents	17 (6.4)	17 (6.3)
Other	5 (1.9)	4 (1.5)
Respiratory failure^c^	32 (12.1)	35 (13.0)
Circulatory failure^c^	1 (0.4)	1 (0.4)
Renal failure^c^	4 (1.5)	5 (1.9)
Triglycerides, mean (SD), mg/dL	1850 (665)	1919 (655)
SIRS score, median (IQR)	2 (1-2)	2 (1-2)
APACHE II score, median (IQR), points	4 (2-6)	4 (2-6)

Abbreviations: AP, acute pancreatitis; APACHE, Acute Physiology and Chronic Health Evaluation; BMI, body mass index (calculated as weight in kilograms divided by height in meters squared); LMWH, low-molecular-weight heparin; SIRS, systemic inflammatory response syndrome.

^a^ This is a referral patient from another hospital.

^b^ Heavy drinker was defined as alcohol consumption of more than 50 g/d for more than 5 years according to the diagnostic criteria of alcoholic pancreatitis; light drinker did not meet the criteria.

^c^ Organ failure included respiratory failure, kidney failure, and circulatory failure, which was defined as a score of 2 or more using the modified Marshall scoring system.

### Primary End Point

In the intention-to-treat analysis, the composite primary end point (new-onset organ failure and/or mortality) occurred in 66 patients (25.0%) in the LMWH plus insulin group compared with 76 patients (28.3%) in the insulin group (RR, 0.89; 95% CI, 0.67-1.17; *P* = .40) (Table 2). No significant between-group difference on the primary end point was observed in the sensitivity analysis adjusted for baseline characteristics (LMWH plus insulin vs insulin alone: adjusted RR, 0.89; 95% CI, 0.67-1.20; *P* = .89) (eTable 4 in Supplement 1) and in the sensitivity analysis among patients who received LMWH for 3 days in the LMWH plus insulin group and those who received insulin alone in the control group (adjusted RR, 0.97; 95% CI, 0.70-1.36; *P* = .87) (eTable 5 in Supplement 1). No significant interaction effects were observed across subgroups stratified by age (<39 vs ≥39 years), baseline AP severity (SIRS score <2 vs ≥2), baseline triglyceride level (<22.6 vs ≥22.6 mg/mL), or duration from pain onset to admission (<24 vs ≥24 hours) (eTable 6 in Supplement 1).

**Table 2.** Primary and Secondary End Points in the Intention-to-Treat Population

Outcome	Patients, No. (%)		RR or MD (95% CI)	*P* value
	LMWH plus insulin group (n = 264)	Insulin group (n = 269)		
A composite of new-onset organ failure and mortality^a^	66 (25.0)	76 (28.3)	0.89 (0.67 to 1.17)	.40
Mortality	1 (0.4)	2 (0.7)	0.51 (0.05 to 5.59)	>.99
New-onset organ failure	66 (25.0)	74 (27.5)	0.91 (0.68 to 1.21)	.51
Respiratory failure	60 (22.7)	69 (25.7)	0.89 (0.66 to 1.20)	.43
Circulatory failure	5 (1.9)	7 (2.6)	0.73 (0.23 to 2.26)	.58
Kidney failure	10 (3.8)	6 (2.2)	1.70 (0.63 to 4.61)	.29
Time to achieve the triglyceride goal of <500 mg/mL, median (IQR), d	2 (1-3)	2 (1-3)	0.00 (0.00 to 0.00)	.94
New-onset SIRS^b^	29 (11.0)	40 (14.9)	0.74 (0.47 to 1.16)	.18
Persistent SIRS^c^	12 (4.5.)	21 (7.8.)	0.58 (0.29 to 1.16)	.12
Pancreatic necrosis	45 (17.0)	49 (18.2)	0.94 (0.65 to 1.35)	.72
Infected pancreatic necrosis	6 (2.3)	6 (2.2)	1.02 (0.33 to 3.12)	.97
Sepsis	4 (1.5)	4 (1.5)	1.02 (0.26 to 4.03)	>.99
Pulmonary infection	9 (3.4)	12 (4.5)	0.76 (0.33 to 1.78)	.53
Abdominal compartment syndrome	2 (0.8)	4 (1.5)	0.51 (0.09 to 2.76)	.69
Portal vein thrombosis	8 (3.0)	10 (3.7)	0.81 (0.33 to 2.03)	.66
Cerebral hemorrhage	0	1 (0.4)	NA	>.99
Mechanical ventilatory support	12 (4.5)	17 (6.3)	0.72 (0.35 to 1.48)	.37
Kidney replacement therapy	5 (1.9)	4 (1.5)	1.27 (0.35 to 4.69)	.75
Percutaneous drainage of ascites	6 (2.3)	10 (3.7)	0.61 (0.23 to 1.66)	.33
Drainage of pancreatic necrosis	6 (2.3)	7 (2.6)	0.87 (0.30 to 2.56)	.81
Necrosectomy of pancreatic necrosis	2 (0.8)	3 (1.1)	0.68 (0.11 to 4.03)	>.99
Vascular interventional therapy	2 (0.8)	1 (0.4)	2.04 (0.19 to 22.34)	.62
ICU stay	40 (15.2)	44 (16.4)	0.93 (0.63 to 1.37)	.70
Length of hospital stay, median (IQR), d	7 (5 to 10)	7 (5 to 11)	0.00 (−1.00 to 0.00)	.54
Hospital costs, median (IQR), ×10^3^ CNY	12 (8 to 19)	11 (7 to 19)	0.38 (−0.83 to 1.50)	.53

Abbreviations: CNY, Chinese yuan; ICU, intensive care unit; LMWH, low-molecular-weight heparin; MD, median difference; NA, not applicable; RR, relative risk; SIRS, systemic inflammatory response syndrome.

^a^ New onset is defined as organ failure not being present for the 24 hours before randomization, and organ failure included respiratory failure, kidney failure, and circulatory failure, which was defined as a score of 2 or more using the modified Marshall scoring system.

^b^ Score of 2 or more using SIRS scoring.

^c^ SIRS persisted for more than 48 hours.

### Secondary End Points

Outcomes of secondary end points are given in Table 2. No significant differences were observed between the groups with regard to mortality (RR, 0.51; 95% CI, 0.05-5.59; *P* > .99), new-onset organ failure (RR, 0.91; 95% CI, 0.68-1.21; *P* = .51), respiratory failure (RR, 0.89; 95% CI, 0.66-1.20; *P* = .43), circulatory failure (RR, 0.73; 95% CI, 0.23-2.26; *P* = .58), or kidney failure (RR, 1.70; 95% CI, 0.63-4.61; *P* = .29). According to the Revised Atlanta Classification^22^ for disease severity stratification, 254 patients (47.7%) were considered to have mild disease, 170 (31.9%) to have moderately severe disease, and 109 (20.5%) to have severe disease. The median (IQR) time to achieve the triglyceride goal of below 500 mg/dL was 2 (1-3) days (*P* = .94) for both groups. More details of the time to achieve the triglyceride goal in the 2 groups are provided in eTable 7 in Supplement 1. Other secondary outcomes, including pancreatic necrosis, infectious complications, abdominal compartment syndrome, cerebral hemorrhage, need for organ support or invasive interventions, length of intensive care unit or hospital stay, and hospital costs, showed no significant intergroup differences. Per-protocol analysis yielded similar results (eTable 8 in Supplement 1).

### Safety Outcomes

New-onset bleeding events occurred in 8 patients: 3 (1.1%) in the LMWH plus insulin group vs 5 (1.9%) in the insulin group (RR, 0.61; 95% CI, 0.15-2.53; *P* = .73). The rebound of triglyceride levels showed a nonsignificant trend toward higher frequency in the insulin group (RR, 0.53; 95% CI, 0.28-1.01; *P* = .050). The incidence of drug-related adverse events did not differ significantly between groups (RR, 0.79; 95% CI, 0.30-2.10; *P* = .64) (Table 3).

**Table 3.** Safety Outcomes in the Intention-to-Treat Population

Outcome	Patients, No. (%)	
	LMWH plus insulin group (n = 264)	Insulin group (n = 269)
New-onset bleeding^a^	3 (1.1)	5 (1.9)
Rebound elevation of serum triglyceride level^b^	13 (4.9)	25 (9.3)
Drugs-related adverse events	7 (2.7)	9 (3.3)
Hypoglycemia	3 (1.1)	4 (1.5)
Local allergic reaction	1 (0.4)	1 (0.4)
Systemic allergic reaction	1 (0.4)	0
Thrombocytopenia	1 (0.4)	0
Elevated transaminases	1 (0.4)	2 (0.7)

Abbreviation: LMWH, low-molecular-weight heparin.

^a^ New-onset bleeding was defined as abdominal or gastrointestinal bleeding occurring within 2 weeks of randomization.

^b^ Levels exceeding those recorded on or before day 3 when measured on or after day 5 after randomization.

## Discussion

This study, to our knowledge, represents the first randomized clinical trial comparing LMWH combined with insulin vs insulin alone in patients with HTG-AP admitted within 48 hours of pain onset. Our results demonstrate that LMWH combined with insulin failed to significantly reduce the incidence of new-onset organ failure and/or mortality compared with insulin alone. Both treatment regimens effectively reduced triglyceride levels with reasonable safety profiles, although more frequent rebound elevation of triglyceride levels was observed in the insulin alone group.

Although insulin has been considered to be the common approach for early triglyceride-lowering therapy in HTG-AP, the unresolved question has been whether the addition of heparin is superior over insulin alone.^29,30^ A previous study^19^ proposed that LMWH may ameliorate AP through enhanced catabolism of triglyceride-rich lipoproteins. However, our study found no significant additional reduction in serum triglyceride levels when LMWH was combined with insulin therapy compared with insulin alone in patients with HTG-AP. The lack of significant difference in serum triglyceride level reduction between groups may partially explain the negative clinical outcomes observed with LMWH treatment.

LMWH has potent anti-inflammatory and anti-oxidative effects, which have been considered to play a role in improving experimental AP.^31,32,33^ In human studies, several evaluating LMWH’s effects on clinical outcomes in AP have reported inconsistent findings.^18,34,35,36^ LMWH was associated with a reduced incidence of multiple organ failure, particularly respiratory failure, in patients with severe AP.^18^ A recent phase 3, single-blind randomized clinical trial demonstrated that LMWH significantly attenuated disease progression and pancreatic necrosis in moderately severe to severe AP.^34^ Other trials failed to demonstrate the differences between the LMWH and control groups.^35,36^ However, in our study, the combination of LMWH and insulin failed to demonstrate significant reductions in new-onset organ failure and/or mortality compared with insulin alone in HTG-AP. The discrepancy between our findings and previous studies^18,34^ favoring LMWH might stem from population heterogeneity in disease severity, which could influence primary outcome incidence. In addition, subgroup analyses demonstrated no significant differences in treatment effects across subgroups stratified by age, AP severity, baseline triglyceride levels, and time from symptom onset to admission. However, in subgroups of younger patients (<39 years) or individuals with baseline triglyceride levels greater than 2000 mg/dL, the point estimate was below 0.75, indicating potential benefits of LMWH therapy in these populations. These results should be interpreted with caution because they were derived from exploratory analyses not adjusted for multiple testing.

### Strengths and Limitations

This study has several notable strengths. Rigorous enrollment protocols ensured timely therapeutic intervention, with a median treatment initiation time of 16 hours after symptom onset. Additionally, the actual event rates for primary outcomes substantially exceeded our pretrial estimation, thereby providing adequate statistical power and minimizing the potential for type 2 error.

The study also has some limitations that warrant consideration. First, the open-label design may have introduced potential observer bias, despite the use of objectively measured clinical end points. Second, the exclusion of patients with triglyceride levels greater than 3550 mg/dL, which was a decision of the ethics committee who think that plasma exchange might be necessary in this population,^37^ may limit the generalizability of our findings to more extreme HTG cases. Third, inflammatory indicators, activity or gene mutation of lipoprotein lipase, and circulating free fatty acids levels were not systematically assessed, precluding analysis of LMWH’s potential effects on these parameters. Fourth, the observed mortality rate was notably low, likely reflecting our exclusion criteria that eliminated high-risk patients (those with triglyceride levels >3550 mg/dL, active bleeding, dialysis-requiring acute kidney failure, irreversible organ failure, or severe chronic comorbidities). Mortality was included due to its indisputable importance and established clinical importance in previous large trials conducted in this population,^38,39,40^ although the low mortality rate makes the trial substantially underpowered for detecting mortality-specific differences.

## Conclusions

In this randomized clinical trial of patients with HTG-AP, those who received LMWH plus insulin therapy demonstrated no incremental clinical benefit compared with insulin monotherapy. These findings indicate that LMWH might not be necessary for early lipid-lowering in HTG-AP.

## Notes

Supplement 1. **eTable 1. **Number of enrolled participants from each participating center **eTable 2.** The dose of enoxaparin patients received on each day **eTable 3.** Missing data of all participants **eTable 4.** Sensitivity analysis of the primary endpoint using robust Poisson regression in all randomized patients **eTable 5.** Sensitivity analysis of the primary endpoint in patients who received LMWH for 3 days in the LMWH + insulin group (n=236) and those who received insulin alone in the control group (n=235) **eTable 6.** Subgroup analysis for the primary endpoint **eTable 7.** The time to achieve the triglyceride goal (< 500 mg/mL) **eTable 8.** Primary and secondary endpoints in the per-protocol population

Supplement 2.
Trial Protocol

Supplement 3. **Nonauthors Collaborators.** Members of the Chinese Acute Pancreatitis Clinical Trials Group (CAPCTG)

Supplement 4.
Data Sharing Statement
