# Supplementary material for: Low-Molecular-Weight Heparin Plus Insulin in Hypertriglyceridemic Acute Pancreatitis: A Randomized Clinical Trial
Source: JAMA Netw Open. 2025 Nov 7;8(11):e2542124. doi: 10.1001/jamanetworkopen.2025.42124 (PMC12595534; doi:10.1001/jamanetworkopen.2025.42124)
Supplement: Supplement 1. — eTable 1. Number of enrolled participants from each participating center eTable 2. The dose of enoxaparin patients received on each day eTable 3. Missing data of all participants eTable 4. Sensitivity analysis of the primary endpoint using robust Poisson regression in all randomized patients eTable 5. Sensitivity analysis of the primary endpoint in patients who received LMWH for 3 days in the LMWH + insulin group (n=236) and those who received insulin alone in the control group (n=235) eTable 6. Subgroup analysis for the primary endpoint eTable 7. The time to achieve the triglyceride goal (< 500 mg/mL) eTable 8. Primary and secondary endpoints in the per-protocol population [file jamanetwopen-e2542124-s001.pdf]

Supplemental Online Content

He W, Ding L, Liu Z, et al; Chinese Acute Pancreatitis Clinical Trials Group (CAPCTG). Low-molecular-weight heparin plus insulin in hypertriglyceridemic acute pancreatitis: a randomized clinical trial. *JAMA Netw Open*. 2025;8(11):e2542124. doi:10.1001/jamanetworkopen.2025.42124

eTable 1. Number of enrolled participants from each participating center
eTable 2. The dose of enoxaparin patients received on each day
eTable 3. Missing data of all participants
eTable 4. Sensitivity analysis of the primary endpoint using robust Poisson regression in all randomized patients
eTable 5. Sensitivity analysis of the primary endpoint in patients who received LMWH for 3 days in the LMWH + insulin group (n=236) and those who received insulin alone in the control group (n=235)
eTable 6. Subgroup analysis for the primary endpoint
eTable 7. The time to achieve the triglyceride goal (< 500 mg/mL)
eTable 8. Primary and secondary endpoints in the per-protocol population

This supplemental material has been provided by the authors to give readers additional information about their work.

© 2025 He W et al. *JAMA Network Open.*

eTable 1. Number of enrolled participants from each participating center

Participating centers	Number of enrolled participants, No.
First Affiliated Hospital of Nanchang University	216
Xinyu People's Hospital	64
Fuzhou First People's Hospital	45
Ganzhou People's Hospital	45
Pingxiang People's Hospital	41
Jingdezhen First People’s Hospital	34
Yingtan People's Hospital	27
Yichun People's Hospital	25
Shangrao People's Hospital	15
First Affiliated Hospital of Shanghai Jiaotong University	8
First Affiliated Hospital of Gannan Medical College	5
Sir Run Run Shaw Hospital	4
First People's Hospital of Yunnan	4

© 2025 He W et al. *JAMA Network Open.*

eTable 2. The dose of enoxaparin patients received on each day

	LMWH + insulin group (n=264)		Insulin alone group (n=269)	
	Enoxaparin IU (average per patient receiving enoxaparin)	N (%) who received No enoxaparin	Enoxaparin IU (average per patient receiving enoxaparin)	N (%) who received No enoxaparin
Day 1	7969.7	1 (0.4)	1011.2	235 (87.4)
Day 2	7969.7	1 (0.4)	951.7	237 (88.1)
Day 3	7969.7	1 (0.4)	921.9	238 (88.5)
Day 4	818.2	237 (89.8)	921.9	238 (88.5)
Day 5	757.6	239 (90.5)	862.5	240 (89.2)
Day 6	636.4	243 (92.0)	803.0	242 (90.0)
Day 7	484.8	248 (93.9)	743.5	244 (90.7)
Day 8	484.8	248 (93.9)	594.8	249 (92.6)
Day 9	181.8	258 (97.9)	297.4	259 (96.3)
Day 10	181.8	258 (97.9)	297.4	259 (96.3)

Abbreviations: LMWH, low-molecular-weight heparin.

© 2025 He W et al. *JAMA Network Open.*

eTable 3. Missing data of all participants

Data	Number of patients with missing data, No. (%)
**Baseline characteristics**	
Sex	0
Age	0
Body-mass index	40 (7.5)
Duration from pain onset to admission	6 (1.1)
Referral	0
History of acute pancreatitis	15 (2.8)
Hypertension	0
Diabetes mellitus	0
Smoker	0
Drinker	0
Hypo-glycaemic agents use before acute pancreatitis onset	0
Hypo-tensive agents use before acute pancreatitis onset	0
Other drugs use before acute pancreatitis onset	0
Respiratory failure	0
Circulatory failure	0
Renal failure	0
Triglyceride level	0
Systemic inflammatory response syndrome score	0
Acute Physiology and Chronic Health Evaluation II score	3 (0.6)
**Primary and secondary endpoints**	
A composite of new-onset OF and mortality	0
Time to achieve the triglyceride goal of < 500 mg/mL	0
New-onset systemic inflammatory response syndrome	0
Pancreatic necrosis	0
Infected pancreatic necrosis	0
Sepsis	0
Pulmonary infection	0
Abdominal compartment syndrome	0
Portal vein thrombosis	0
Cerebral haemorrhage	0
Mechanical ventilation	0
Renal replacement therapy	0
Percutaneous drainage of ascites	0
Drainage of pancreatic necrosis	0
Necrosectomy of pancreatic necrosis	0
Vascular interventional therapy	0

© 2025 He W et al. *JAMA Network Open.*

ICU stay	0
Length of hospital stay	3 (0.6)
Hospital costs	12 (2.3)
**Safety outcomes**	
New-onset bleeding	0
Rebound elevation of serum triglyceride	0
Drugs-related adverse events	0
Hypoglycaemia	0
Local allergic reaction	0
Systemic allergic reaction	0
Thrombocytopenia	0
Elevated transaminases	0

© 2025 He W et al. *JAMA Network Open.*

eTable 4. Sensitivity analysis of the primary endpoint using robust Poisson regression in all randomized patients

Variables	Adjusted RR (95% CI)[a]	*P* value
Treatments		
Insulin group	Reference	Reference
LMWH + insulin group	0.89 (0.67-1.20)	.89
Sex		
Male	Reference	Reference
Female	0.81 (0.53-1.24)	.33
Age	0.99 (0.98-1.01)	.37
Body-mass index	1.01 (0.97-1.05)	.61
Duration from pain onset to admission	1.00 (0.98-1.01)	.71
Referral		
No	Reference	Reference
Yes	1.38 (0.98-1.96)	.07
History of AP	0.88 (0.73-1.07)	.21
Hypertension		
No	Reference	Reference
Yes	1.17 (0.71-1.93)	.55
Diabetes mellitus		
No	Reference	Reference
Yes	1.21 (0.80-1.82)	.37
Smoker		
Never	Reference	Reference
Current	1.10 (0.74-1.63)	.65
Past	2.73 (1.14-6.56)	.03
Drinker		
Never	Reference	Reference
Light	0.95 (0.62-1.45)	.82
Heavy	0.69 (0.26-1.82)	.69
Hypo-glycaemic agents use before AP onset		
No	Reference	Reference
Yes	0.97 (0.57-1.66)	.91
Hypo-tensive agents use before AP onset		
No	Reference	Reference
Yes	1.07 (0.48-2.40)	.87
Other drugs use before AP onset		
No	Reference	Reference
Yes	2.51 (1.43-4.40)	.001
Respiratory failure		

© 2025 He W et al. *JAMA Network Open.*

No	Reference	Reference
Yes	0.21 (0.10-0.44)	<.001
Circulatory failure	NA	NA
Renal failure		
No	Reference	Reference
Yes	0.85 (0.31-2.34)	.75
TG	1.03 (1.01-1.05)	.02
SIRS score	1.10 (0.93-1.31)	.28
APACHE II score	1.10 (1.05-1.14)	<.001

Abbreviations: RR, relative risk; CI, confidence interval; LMWH, low-molecular-weight heparin; AP, acute pancreatitis; TG, triglyceride; SIRS, systemic inflammatory response syndrome; APACHE, Acute Physiology and Chronic Health Evaluation.

[a] using Poisson regression with robust standard error estimation.

© 2025 He W et al. *JAMA Network Open.*

eTable 5. Sensitivity analysis of the primary endpoint in patients who received LMWH for 3 days in the LMWH + insulin group (n=236) and those who received insulin alone in the control group (n=235)

Variables	Adjusted RR (95% CI)[a]	*P* value
Treatments		
Insulin group	Reference	Reference
LMWH + insulin group	0.97 (0.70-1.36)	.87
Sex		
Male	Reference	Reference
Female	0.79 (0.48-1.30)	.35
Age	0.99 (0.97-1.01)	.56
Body-mass index	1.01 (0.97-1.05)	.57
Duration from pain onset to admission	0.99 (0.98-1.01)	.48
Referral		
No	Reference	Reference
Yes	1.39 (0.93-2.09)	.11
History of AP	0.88 (0.69-1.13)	.32
Hypertension		
No	Reference	Reference
Yes	1.28 (0.76-2.16)	.35
Diabetes mellitus		
No	Reference	Reference
Yes	1.35 (0.86-2.13)	.20
Smoker		
Never	Reference	Reference
Current	1.13 (0.71-1.81)	.60
Past	3.09 (1.27-7.55)	.01
Drinker		
Never	Reference	Reference
Light	0.95 (0.57-1.57)	.83
Heavy	0.80 (0.30-2.10)	.64
Hypo-glycaemic agents use before AP onset		
No	Reference	Reference
Yes	0.87 (0.48-1.57)	.63
Hypo-tensive agents use before AP onset		
No	Reference	Reference
Yes	1.18 (0.52-2.68)	.70
Other drugs use before AP onset		
No	Reference	Reference

© 2025 He W et al. *JAMA Network Open.*

Yes	2.77 (1.50-5.14)	.001
Respiratory failure		
No	Reference	Reference
Yes	0.23 (0.10-0.51)	<.001
Circulatory failure	NA	NA
Renal failure		
No	Reference	Reference
Yes	0.94 (0.26-3.41)	.92
TG	1.03 (1.00-1.05)	.04
SIRS score	1.12 (0.92-1.37)	.27
APACHE II score	1.09 (1.03-1.14)	.002

Abbreviations: LMWH, low-molecular-weight heparin; RR, relative risk; CI, confidence interval; AP, acute pancreatitis; TG, triglyceride; SIRS, systemic inflammatory response syndrome; APACHE, Acute Physiology and Chronic Health Evaluation.

[a] using Poisson regression with robust standard error estimation.

eTable 6. Subgroup analysis for the primary endpoint

	Patients, No. (%)			
Variables	LMWH + insulin group	Insulin group	RR (95% CI)	P value for interaction
Age				.18
<39 y	29 (22.8)	42 (31.3)	0.73 (0.49-1.09)	
≥39 y	37 (27.0)	34 (25.2)	1.07 (0.72-1.60)	
Baseline AP severity				.71
SIRS score <2 points	21 (17.9)	28 (22.4)	0.80 (0.48-1.33)	
SIRS score ≥2 points	45 (30.6)	48 (33.3)	0.92 (0.66-1.29)	
Baseline TG level strata				.28
TG <2000 mg/dL	43 (24.0)	39 (23.5)	1.02 (0.70-1.49)	
TG ≥2000 mg/dL	23 (27.1)	37 (35.9)	0.75 (0.49-1.16)	
Duration from pain onset to admission				.99
<24 hours	39 (23.5)	42 (26.4)	0.89 (0.61-1.30)	
≥24 hours	27 (27.6)	34 (30.9)	0.89 (0.58-1.36)	

LMWH, low-molecular-weight heparin; RR, relative risk; CI, confidence interval; AP, acute pancreatitis; SIRS, systemic inflammatory response syndrome; TG, triglyceride.

© 2025 He W et al. *JAMA Network Open.*

eTable 7. The time to achieve the triglyceride goal (< 500 mg/mL)

	Patients, No. (%)	
Days after randomization	LMWH + insulin group (n=264)	Insulin group (n=269)
Day 1	67 (25.4)	64 (23.8)
Day 2	47 (17.8)	69 (25.7)
Day 3	38 (14.4)	39 (14.5)
Day 5	31 (11.7)	32 (11.9)
Day 7	13 (4.92)	14 (5.20)
Day 14	3 (1.14)	2 (0.74)
NA[a]	65 (24.6)	49 (18.2)

Abbreviations: LMWH, low-molecular-weight heparin.

[a] Not achieve the goal during observation.

© 2025 He W et al. *JAMA Network Open.*

eTable 8. Primary and secondary endpoints in the per-protocol population

	Patients, No. (%)			
Outcome	LMWH + insulin group (n=247)	Insulin group (n=216)	RR/MD (95% CI)	*P* value
A composite of new-onset OF and mortality[a]	60 (24.3)	53 (24.5)	0.99 (0.72-1.37)	.95
Mortality	0	1 (0.5)	NA	.47
New-onset OF	60 (24.3)	52 (24.1)	1.01 (0.73-1.39)	.96
New-onset respiratory failure	56 (22.7)	49 (22.7)	1.00 (0.71-1.40)	>.99
New-onset circulatory failure	2 (0.8)	3 (1.4)	0.58 (0.10-3.46)	.67
New-onset renal failure	8 (3.2)	2 (0.9)	3.50 (0.75-16.30)	.11
Time to achieve the TG goal of < 500 mg/mL, median (IQR), days	2 (1-3)	2 (1-3)	0.00 (0.00-0.00)	.74
New-onset SIRS[b]	28 (11.3)	28 (13.0)	0.87 (0.54-1.43)	.59
New-onset persistent SIRS[c]	11 (4.5)	12 (5.6)	0.80 (0.36-1.78)	.59
Pancreatic necrosis	41 (16.6)	37 (17.1)	0.97 (0.65-1.45)	.88
Infected pancreatic necrosis	4 (1.6)	1 (0.5)	3.50 (0.39-31.06)	.38
Sepsis	4 (1.6)	0	NA	.13
Pulmonary infection	8 (3.2)	5 (2.3)	1.40 (0.47-4.21)	.55
Abdominal compartment syndrome	2 (0.8)	0	NA	.50
Cerebral hemorrhage	0	0	NA	NA
Mechanical ventilation	10 (4.0)	6 (2.8)	1.46 (0.54-3.94)	.46
Renal replacement therapy	3 (1.2)	0	NA	.25

© 2025 He W et al. *JAMA Network Open.*

Percutaneous drainage of ascites	5 (2.0)	4 (1.9)	1.09 (0.30-4.02)	>.99
Drainage of pancreatic necrosis	4 (1.6)	2 (0.9)	1.75 (0.32-9.46)	.69
Necrosectomy of pancreatic necrosis	1 (0.4)	1 (0.5)	0.87 (0.06-13.90)	>.99
Vascular interventional therapy	1 (0.4)	0	NA	>.99
ICU stay	35 (14.2)	20 (9.3)	1.53 (0.91-2.57)	.10
Length of hospital stay, median (IQR), days	7 (5-10)	7 (5-10)	0.00 (-1.00-1.00)	.98
Hospital costs, median (IQR), x10^3 CNY	12 (8-18)	10 (7-16)	0.80 (-0.32-1.97)	.15

Abbreviations: LMWH, low-molecular-weight heparin; RR, relative risk; MD, median difference; CI, confidence interval; OF, organ failure; TG, triglyceride; IQR, interquartile range; SIRS, systemic inflammatory response syndrome; ICU, intensive care unit; CNY, Chinese yuan.

a New onset is defined as organ failure not being present for the 24 hours prior to randomization, and organ failure included respiratory failure, renal failure, and circulatory failure, which was defined as a score of 2 or more using the modified Marshall scoring system.

b SIRS was a score of 2 or more using SIRS score.

c Persistent meant SIRS persisted for more than 48 hours.

© 2025 He W et al. *JAMA Network Open.*
