# Supplementary material for: Low-Molecular-Weight Heparin Plus Insulin in Hypertriglyceridemic Acute Pancreatitis: A Randomized Clinical Trial
Source: JAMA Netw Open. 2025 Nov 7;8(11):e2542124. doi: 10.1001/jamanetworkopen.2025.42124 (PMC12595534; doi:10.1001/jamanetworkopen.2025.42124)
Supplement: Supplement 3. — Nonauthors Collaborators. Members of the Chinese Acute Pancreatitis Clinical Trials Group (CAPCTG) [file jamanetwopen-e2542124-s003.pdf]

**CLINICAL STUDY PROTOCOL**

# Low-molecular-weight heparin plus insulin versus insulin alone for hypertriglyceridemia-associated acute pancreatitis: protocol of a multicenter, open-label, randomized, controlled trial

**Version 2.0 (June 5, 2019)**

**Principal investigator: Nonghua Lv , First Affiliated Hospital, Jiangxi Medical College, Nanchang University**

## SUMMARY OF THE TRIAL

| | |
|---|---|
| **Title** | Low-molecular-weight heparin plus insulin versus insulin alone for hypertriglyceridemia-associated acute pancreatitis: protocol of a multicenter, open-label, randomized, controlled trial |
| **Study design** | Multi-center, open-label, randomized, controlled trial |
| **Primary objective** | To compare the efficacy and safety of Low-molecular-weight heparin (LMWH) plus insulin versus insulin alone in reducing the incidence of new-onset organ failure and/or mortality among patients with hypertriglyceridemia-associated acute pancreatitis (HTG-AP). |
| **Study center(s)** | Up to 10 study centers |
| **Number of subjects (planned)** | Approximately 540 randomized subjects |
| **Subjects** | **Inclusion criteria**<br>● Patients aged between 18 and 85 years;<br>● Acute pancreatitis diagnosed according to the 2012 revised Atlanta Classification;<br>● Time from acute pancreatitis onset to randomization within 48 hours;<br>● Serum triglyceride levels ranged from 11·3 mmol/L (equal to 1000 mg/dL) to 40 mmol/L (equal to 3550 mg/dL);<br>● Signed informed consent.<br><br>**Exclusion criteria**<br>● Biliary pancreatitis;<br>● Alcoholic pancreatitis;<br>● Pregnant or lactating females; |

| | |
|---|---|
| | ● Disseminated intravascular coagulation or active bleeding;<br>● Allergy to heparin or insulin;<br>● Usage of any triglyceride-lowering treatments between symptoms onset and randomization, or blood purification treatment for acute renal failure;<br>● Cardiopulmonary resuscitation for cardiac arrest;<br>● Patients with irreversible organ failure or other critical manifestations who are expected to die within 24 hours;<br>● Advanced malignant tumors, immunodeficiency disorders, and a pre-existing chronic renal failure requiring regular hemodialysis;<br>● Conscious and behavioral abilities are partially or totally limited and unable to make decisions independently. |
| **Treatments** | **LMWH + insulin group**<br>a 4000 IU of LMWH sodium injection (enoxaparin) will be injected subcutaneously once every 12 hours for three days. Insulin adjusted on the glucose levels.<br>**Insulin alone group**<br>Insulin adjusted on the glucose levels. |
| **Duration of Treatment** | Three days for LMWH. |
| **Follow-up** | All participants will be followed for a total of one month. |
| **Informed consent** | Written informed consent will be obtained from all participants or their legal representatives. |
| **Randomization** | Patients will be randomly assigned (1:1) to either LMWH plus insulin (LMWH + insulin group) or insulin alone (insulin group). Dynamic randomization will be used via a network-based center random software. |

| | |
|---|---|
| **Primary outcome** | A composite of new-onset organ failure and/or mortality within one month after randomization |
| **Secondary outcomes** | 1) New-onset organ failure including respiratory failure, renal failure, and circulatory failure; 2) Mortality; 3) The time to achieve the triglyceride goal of <5·65 mmol/L, which will be detected using a fully automated blood biochemistry analyser; 4) New-onset systematic inflammatory response syndrome; 5) Necrotizing pancreatitis; 6) Infected pancreatic necrosis; 7) Sepsis; 8) Pulmonary infection; 9) Vein thrombosis; 10 ) Pseudoaneurysm; 11) Sinistral portal hypertension; 12) Abdominal compartment syndrome; 13) Cerebral haemorrhage; 14) Demand for mechanical ventilation; 15) Renal replacement therapy; 16) Percutaneous drainage of ascites; 17) Drainage of pancreatic necrosis; 18) Debridement of pancreatic necrosis; 19) Vascular interventional therapy; 20) Duration of intensive care unit stay; 21) Length of hospital stay; 22) Hospital costs. |
| **Safety outcomes** | new-onset bleeding, the rebound of serum triglyceride, and drug-related adverse events. |
| **Study Timeline** | Recruitment period estimated in 36 months<br>Follow-up per patient: one month |

**PROTOCOL SIGNATURE PAGE PRINCIPAL INVERTIGATOR**

**Study titled: Low-molecular-weight heparin plus insulin versus insulin alone for hypertriglyceridemia-associated acute pancreatitis: protocol of a multicenter, open-label, randomized, controlled trial**

**The signatures on this page indicate review and approval of the final version of the protocol.**

**By signing this document, we confirm that the clinical study will be conducted in accordance with the protocol and all applicable laws and regulations including, but not limited to, the International Conference on Harmonization Guideline for Good Clinical Practice (GCP) and the ethical principles that have their origins in the Declaration of Helsinki.**

| | |
|---|---|
| **Signature:** | **June 5, 2019** |
| **Professor Nonghua Lv** | **Date of signature** |
| **Principal investigator** | |

## 1 INTRODUCTION

Acute pancreatitis (AP) is a common gastrointestinal disease with multiple causes, including gallstones, alcohol, and hypertriglyceridemia (HTG)[1]. Basolateral secreted or leaked pancreatic lipase-mediated triglyceride (TG) lipolysis may play an important role in the initiation and exacerbation of hypertriglyceridemia-associated acute pancreatitis (HTG-AP). This lipolysis process leads to the accumulation of free fatty acids (FFAs), which can cause cytotoxic injury to the pancreas[2-6]. Notably, increased serum TG levels have been linked to worse clinical outcomes, manifesting as persistent systemic inflammatory response syndrome (SIRS) and organ failure[7-11].

On that basis, there have been some treatment strategies introduced to reduce serum TG levels, including noninvasive measures such as fasting, insulin, and heparin, as well as invasive blood purification techniques[12]. Despite these interventions, international guidelines for the early management of AP did not give any recommendations regarding specific TG-lowering therapy due to the lack of solid evidence[13-15]. Nonetheless, in some clinical settings, insulin with or without heparin has emerged as a common therapeutic approach for the early management of HTG-AP[16]. Heparin, including low-molecular-weight heparin (LMWH), stimulates the release of endothelial lipoprotein lipase (LPL) into circulation, while insulin activates LPL and reduces the activity of hormone-sensitive lipase, thereby increasing the clearance of chylomicrons from plasma[17-20].

Although heparin with insulin is commonly used for lowering-TG in the treatment of HTG-AP, the efficacy and safety of LMWH plus insulin in HTG-AP remains controversial. Our previous RCT showed that LMWH plus insulin not only rapidly decreased the TG levels within 48 hours, but also significantly decreased the incidence of respiratory failure in HTG-AP patients[16]. Additionally, heparin might reduce pancreatic necrosis and improve organ function, by its anti-inflammation and antioxidative properties[21]. However, the bleeding risk of low-molecular-weight heparin (LMWH) was significantly lower than that of unfractionated heparin[22], and it exerts

anti-inflammatory effects[23]. A multicenter prospective study found that LMWH can reduce the incidence of complications, mortality and hospital stay in severe acute pancreatitis (SAP), and no haemorrhagic complications occurred[24]. However, it was concerned that prolonged use of heparin might lead to a rebound of serum TG due to the LPL depletion[25-27], and increase the risk of pancreatic hemorrhage. However, our previous RCT showed that a short course of LMWH use for three days did not induce the rebound of TG or any serious bleeding event[16]. In this multicenter RCT, we will compare the efficacy and safety of LMWH plus insulin versus insulin alone in reducing the incidence of new-onset organ failure and mortality in patients with HTG-AP.

## 2 TRIAL OBJECTIVES

To compare the efficacy and safety of LMWH plus insulin versus insulin alone in reducing the incidence of new-onset organ failure and/or mortality in patients with HTG-AP.

## 3 OVERALL STUDY DESIGN

### 3.1 Study design

This is an investigator-initiated, multicenter, open-label, parallel, randomized, controlled trial.

### 3.2 Study population

Adult patients admitted to any hospital of the participating hospitals with the primary diagnosis of AP meeting the inclusion criteria will be screened for inclusion into the study.

### 3.3 Inclusion criteria

- Patients aged between 18 and 85 years;
- AP diagnosed according to the 2012 revised Atlanta Classification[28];
- Time from AP onset to randomisation within 48 hours;
- Serum TG levels ranged from 11·3 mmol/L (equal to 1000 mg/dL) to 40 mmol/L (equal to 3550 mg/dL);

- Signed informed consent.

**3.4 Exclusion criteria**

- Biliary pancreatitis defined as the presence of dilated choledocholith, or meeting two of the following three laboratory abnormalities: (1) serum bilirubin concentration >1.9 mg/dL; (2) alanine aminotransferase (ALT) activity >100 U/L with ALT activity higher than the aspartate aminotransferase (AST) activity; and (3) alkaline phosphatase activity >195 U/L with γ-glutamyltransferase (GGT) activity >45 U/L;
- Alcoholic pancreatitis defined as a history of over 5 years of heavy alcohol consumption (>50 g per day);
- Pregnant or lactating females;
- Disseminated intravascular coagulation or active bleeding;
- Allergy to heparin or insulin;
- Usage of any TG-lowering treatments (including TG-lowering drugs, plasma exchange, or haemofiltration) between symptoms onset and randomisation, or blood purification treatment for acute renal failure;
- Cardiopulmonary resuscitation for cardiac arrest;
- Patients with irreversible organ failure or other critical manifestations who are expected to die within 24 hours, including patients with severe ARDS requiring extracorporeal life support ($FiO_2/PO_2$ <60 if PEEP >10 cm $H_2O$ and $FiO_2$ =100%), severe systemic circulatory failure (a systolic blood pressure <90 mmHg and serum pH values <7.0 given full-fluid resuscitation and norepinephrine usage at a dose of 25 μg/min), or severe coma without sedation drugs with a Glasgow score equal to three points;
- Advanced malignant tumours, immunodeficiency disorders, and a pre-existing chronic renal failure requiring regular haemodialysis;
- Conscious and behavioural abilities are partially or totally limited and unable to make decisions independently.

## 4 STUDY PROCEDURES

### 4.1 Randomization and masking

Patients will be randomly assigned (1:1) to either LMWH plus insulin (LMWH + insulin group) or insulin alone (insulin group). Dynamic randomization will be used via a network-based center random software (Churun Information Technology Co, Ltd, Guangzhou, China). The randomization and masked allocation assignments will be conducted by the sub-investigator from each entity.

### 4.2 Blinding

This study is an open-label study, and both participants and investigators will not be blinded to the study.

### 4.3 Intervention Group (LMWH + insulin group)

In the LMWH + insulin group, a 4000 IU of LMWH sodium injection (enoxaparin) will be injected subcutaneously once every 12 hours for three days[16, 27]. Continuous usage of LMWH at prophylactic dosages will be considered for patients assessed to be at high-highest risk of venous thromboembolism and low bleeding risk, which was on chief physician's discretion[29].

For those with a glucose level > 11·1 mmol/L, patients will receive micropumps of insulin at 2 to 6 U/h[30, 31], monitor blood glucose closely, to maintain the serum glucose levels between 7·8 and 10 mmol/L[32]. When the glucose levels fall below 7·8 mmol/L, 6 U of insulin will be added to 500 mL of 5% glucose for intravenous infusion until the TG levels decrease to less than 5·65 mmol/L (500 mg/dL) or for a maximum duration of 5 days after randomization, whichever occurs first. For those with a glucose level <=11.1 mmol/L, patients will receive 6 U of insulin with 500 mL of 5% glucose for intravenous, twice a day for 5 days, if hypoglycemia occurs, 5% dextrose change to 10% dextrose.

### 4.4 Control Group (insulin group)

The insulin group received identical insulin therapy as described for the LMWH + insulin group.

### 4.5 Drug Withdrawal Standard

When TG levels reach less than 500 mg/dL or at 5 days after enrollment, insulin will

be stopped and oral lipid-lowering drugs will be administered in the above two groups. If the blood glucose level is still greater than 11.1 mmol/L, insulin will be maintained to control the blood sugar. While LMWH administration was limited to 3 days (irrespective of triglyceride levels) in the LMWH + Insulin Group. However, LMWH (4000 IU every 12 hours for 7-10 days) was administered as venous thromboembolism (VTE) prophylaxis in high-risk patients (Padua score ≥4) ,and with low bleeding risk in both groups[23].

**4.6 General Treatment Regimen**

Early medical treatments include fasting for 48 hours, fluid resuscitation and analgesia. Patients will be offered intensive care after organ failure. Antibiotics were given after clinically suspected or confirmed infection. Mild HTGP patients without severe abdominal pain or vomiting after admission will be orally fed low-fat food. Moderate and severe HTGP patients who cannot tolerate oral feeding will be given a nasojejunal nutrition tube for enteral nutrition. Enteral nutrition used by nasal jejunal tube is generally started at 72 hours after AP onset with a low dose of peptison (20 mL/h). The nutritional goal is achieved after 1 week if the tolerance of enteral nutrition feeding is gradually increased. The patient would reduce the dose of enteral nutrition, and the enteral nutrition feeding would generally be stopped 1 month later. Patients with serum TG levels of greater than 500 mg/dL will not be given enteral nutrient solutions containing fatty milk. If the TG levels remain at 250 mg/dL at 5 days after enrollment, oral lipid-lowering drugs will be administered, and 200 mg/d of fenofibrate (Recipharm, Fontaine, France) is the first choice for the long-term control of TG levels. Serum TG levels will be controlled with a combination of statin lipid-lowering drugs if they cannot be controlled by fibrates alone.

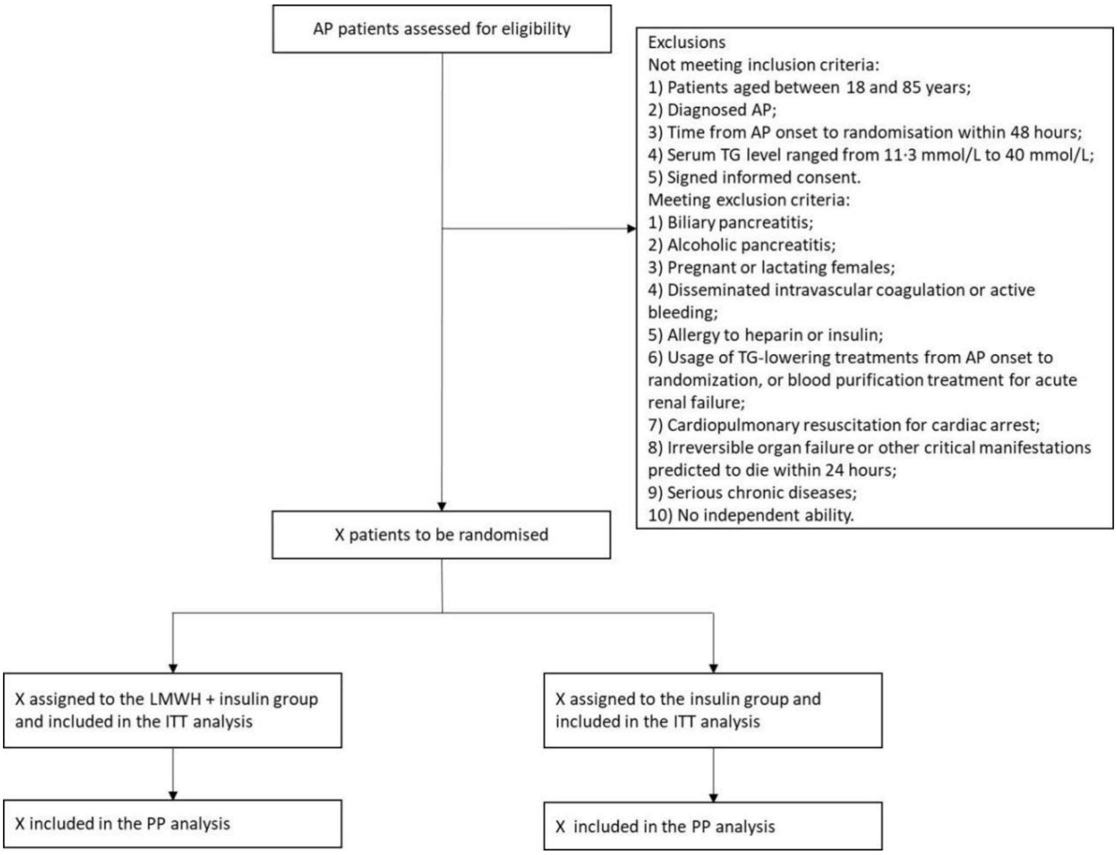


**Figure 1** Flow chart of the study. AP, acute pancreatitis; TG, triglyceride; LMWH, low-molecular-weight heparin; ITT, intention to treat; PP, per-protocol.

## 5 OUTCOMES

### 5.1 Primary endpoint

The primary endpoint is a composite of new-onset organ failure and mortality within one month after randomization, excluding events occurring within 24 hours before randomization. Organ failure includes respiratory failure, renal failure, and circulatory failure, which is defined as a score of 2 or more using the modified Marshall scoring system[28].

### 5.2 Secondary endpoints

All the endpoints are assessed and collected within one month after randomization including 1) New-onset organ failure including respiratory failure, renal failure, and circulatory failure; 2) Mortality; 3) The time to achieve the TG goal of <5·65 mmol/L, which will be detected using a fully automated blood biochemistry analyzer; 4) New-onset SIRS; 5) Necrotizing pancreatitis; 6) Infected pancreatic necrosis; 7) Sepsis;

8) Pulmonary infection; 9) Vein thrombosis; 10 ) Pseudoaneurysm; 11) Sinistral portal hypertension; 12) Abdominal compartment syndrome; 13) Cerebral hemorrhage; 14) Demand for mechanical ventilation; 15) Renal replacement therapy; 16) Percutaneous drainage of ascites; 17) Drainage of pancreatic necrosis; 18) Debridement of pancreatic necrosis; 19) Vascular interventional therapy; 20) Duration of intensive care unit stay; 21) Length of hospital stay; 22) Hospital costs.

**5.3 Safety endpoints**

Safety outcomes include the new-onset bleeding, the rebound of serum TG, and drug-related adverse events (AEs). The new-onset bleeding is defined as new-onset abdominal or gastrointestinal bleeding occurring within two weeks after randomization. The rebound of TG is indicated by an increase in TG levels after prolonged use of heparin, with TG levels measured five days after randomization surpassing the TG levels measured within three days after randomization.

| | STUDY PERIOD | | | | | | | |
|---|---|---|---|---|---|---|---|---|
| | Enrolment | Allocation | Post-allocation | | | | | Close-out |
| TIMEPOINT | Admission | Day 0 | Day 1 | Day 2 | Day 3 | Day 4 | Day 5 | Day 28 |
| ENROLMENT | | | | | | | | |
| Eligibility screen | X | | | | | | | |
| Informed consent | X | | | | | | | |
| Allocation | | X | | | | | | |
| INTERVENTION | | | | | | | | |
| Two groups | | | X | X | X | X | X | |
| ASSESSMENTS | | | | | | | | |
| Baseline variables | X | X | | | | | | |
| Primary endpoint | | | | | | | | X |
| Secondary endpoints | | | X | X | X | X | X | X |
| Safety endpoints | | | X | X | X | X | X | X |

**Figure 2** The schedule of enrolment, interventions, and assessments of the study.

## 6 ETHICS AND DISSMINATION

### 6.1 Guiding principles

This study is to be performed in accordance with the ethical principles of the

Declaration of Helsinki (June 1964 and amended 1975, 1983, 1989, 1996, 2000, 2008, 2013 and Note of Clarification 2002 and 2004), China GCP Notes for Guidance on Good Clinical Practice (GCP).

**6.2 Ethical considerations**

Ethical review procedure: All subsites engaged in this study must be reviewed and approved by the ethics committee of the hospital before study initiation. The amendment of the study protocol and informed consent form should be submitted to the ethics committee for re-review and re-approval;

Ethics committee review of the sponsor's organization: Prior to study initiation, the principal investigator should submit the investigator's brochure, study protocol, informed consent form, CRF, and qualifications and resumes of the investigators, and other major study materials to the ethics committee of the study sponsor's organization for review and approval;

Ethics committee review of the subsite organization: The principal investigator of each subsite should submit the approval letter issued by ethics committee of the sponsor's organization, the investigator's brochure, study protocol, informed consent form, CRF, qualifications and resumes of the principal investigator from the subsite, and other study data to the ethics committee of the hospital for review and approval;

When reviewing and approving study documents, the ethics committee must confirm the study title and indicate the version number, version date and review date of the reviewed study documents.

**6.3 Informed consent form**

The investigator must inform the subject or his/her legal representative of the information about the study in both oral and written ways. The subjects or their legal representatives have the right to know the detailed information about this study.

The informed consent form (ICF) must be reviewed and approved by the ethics committee. If necessary, the investigator is responsible for explaining the contents of the ICF to the subject in a manner that the subject can understand. The subject or his/her legal representative should have sufficient time to read and understand the

ICF before signing it.

The ICF must be signed and dated by the subject or his/her legal representative. The signed ICF should be kept separately by the investigator and the subject, and the investigator should keep the original copy on file for monitoring and audit.

**6.4 Privacy Protection of Subjects**

The investigator takes the responsibility to maintain the anonymity of subjects and keep documents showing the identity of subjects strictly confidential. In CRFs or other study documents, subjects can only be identified with codes. The investigator must properly maintain a screening form that records the subject code, name, and home address.

**6.5 Dissemination Policy**

A writing committee will be formed to review and publish the data from the study. This committee will consist of the Steering Committee and a subset of investigators. The writing committee will write/review all drafts of abstracts and full-length manuscripts and will choose the appropriate journal (for manuscripts) or meeting (for abstracts) for submission.

The steering committee commits that when the study is completed, the data from this study will be published, regardless of the outcome of the study. All information concerning the trial supplied to the investigators by the steering committee and not previously published is considered confidential and shall remain the sole property of the steering committee. The investigator agrees to use this information only in accomplishing the study and will not use it or the data generated from the study for other purposes without first obtaining written authorization from the steering committee.

## 7 DATA MANAGEMENT

All data will be collected prospectively using paper-based case report forms (CRFs). Data entry was performed by the nominated investigators (one or two in each participating center). The quality of CRFs was regularly (almost 6 months) inspected by independent investigators to ensure accuracy, completeness and legibility. Any

missing, implausible, or inconsistent data recorded in the CRFs were sent back to investigators to review and amend if necessary and before clean file status was declared.

An electronic database (EpiData Entry, https://www.epidata.dk/) was used for data double entry and validation. All data stored in the electronic database were de-identified to ensure patient confidentiality. Training for data entry and the use of the EpiData software was performed by the lead site (FAHNU), which also assumed responsibility for data safety, privacy and quality.

## 8 STATISTICAL CONSIDERATIONS

### 8.1 Sample size

This study is designed as a superiority trial, and the sample size is calculated based on data from previous clinical studies[16]. The occurrence rate of the new-onset organ failure and/or mortality of LMWH combined with insulin in HTGP patients is 8.3%, whereas that for treatment with insulin alone in HTGP patients is 17.86%. We estimate that a sample size of 490 participants will provide 80% power at a two-sided alpha level of 0·05 to detect a 9.56% reduction in the composite primary endpoint. Given an attrition rate of 10%, we plan to randomize 540 patients in total (270 per group). The sample size was calculated using PASS software (PASS V.11, NCSS software, Kaysville, USA).

### 8.2 Statistical analysis

Three populations will be defined:

- Intention to treat (ITT) population: the set of all randomized participants.
- Per-protocol (PP) population: the set of all randomized participants, excluding cases with protocol violation, or withdrawal on patients willing.
- Safety analysis population: the set of all randomized participants.

Baseline variables will be presented as median with 25$^{th}$-75$^{th}$ percentile or frequencies with percentages. The primary endpoint will be analyzed using the Chi-Squared Test. The secondary endpoints and safety outcomes will be compared by the Chi-Squared Test, Fisher's exact test, or Wilcoxon rank-sum test, as appropriate.

Results will be presented as relative risk (RR) with 95% confidence intervals (CIs). All statistical analyses will be performed using SPSS software (v26.0; SPSS Inc., Chicago, IL, USA), with significance set at a two-sided α level of 5%.

### 8.3 Premature termination of the study

No interim analysis will be performed. In this trial, the IDMC will examine safety variables every six months. The TMC will discuss, and if the adverse effects are confirmed, they will be reported to the medical ethic committee of the FAHNU. The medical ethic committee of the FAHNU will decide whether to terminate the trial, and audit trial conduct every 12 months independent from investigators.

## 9 SAFETY MONITORING AND MANAGEMENT

### 9.1 Trial management committee

This trial will be coordinated by the trial management committee (TMC), which is formed comprising the principal investigator and the sub-investigators from all the participating centres. The TMC will be responsible for the day-to-day running, coordinating, and management of the trial. An expert clinical panel including members from each of the participating sites is formed to provide governance and audition for the study. This panel also will assist with making major clinical decisions.

### 9.2 Independent Data Monitoring Committee

The independent Data Monitoring Committee (IDMC) will manage data, data security and monitoring. An Independent Data and Safety Monitoring Committee (IDSMC) who are responsible for overseeing the safety and quality of the data. The committee is independent and has no competing interests with the research. The IDSMC considered protocol adherence, trial withdrawal, and safety monitoring and made recommendations for continuation of the trial.

### 9.3 Safety

Insulin with or without heparin is regarded as safe therapy approach in routine clinical settings. Given the known safety endpoints mentioned in the Outcomes section and the absence of unknown drugs or therapies in the study, it is not anticipated that any further AEs or serious AEs would occur or be attributable to the

intervention during the trial. Throughout this trial, the IDMC will examine safety variables every six months. Investigators will also report serious AEs on a separate form, which must be sent to the IDMC and the medical ethics committee of the FAHNU.

## 10 Discussion

This study was the first randomized trial comparing LMWH plus insulin versus insulin alone in patients with HTG-AP admitted within 48 hours. The initial version of the study protocol was a multicenter, prospective, single-blind, randomized controlled trial, which was published in Pancreas.[31] However, before the study was initiated, the protocol was revised after all the research centers discussed it: firstly, the ethical committees at some centers reviewed that LMWH was also needed for patients entering the insulin group who were at very high risk of thrombosis. In addition, patients at 48 hours of onset endured pain, and the additional subcutaneous injection of placebo (saline) in the control group added to patient suffering, so changing from single-blind to open-label was recommended due to poor feasibility; secondly, statisticians recommended revising the sample size from 476 to 540 cases based on the calculations of a superiority trial; thirdly, experts recognized that acute renal failure increases the risk of hemorrhage with LMWH, and that these patients need to undergo blood purification, which affects TG levels, so they were excluded. Finally, we retracted the original protocol after discussion, and the revised protocol was approved by the Ethics Committees and updated in the Clinical Trial Registry(https://www.chictr.org.cn/showproj.html?proj=28529).

The reason for placing an upper limit on TG levels will be based on the ethics committee's advise; prior to the initiation of this study, several research centers routinely placed patients with TG >3550 mg/dL on plasma exchange in clinical practice, as it has been reported that hemodialysis of patients with extremely high levels of serum TG shortens the length of hospital stay[32].

## 11 Funding

None of the funding organizations will contribute to the study design; collection, management, analysis and interpretation of data; writing of the report or the decision to submit the report for publication.

## REFERENCES

1. Forsmark CE, Vege SS, Wilcox CM. Acute Pancreatitis. N Engl J Med 2016;375:1972-1981.
2. de Oliveira C, Khatua B, Bag A, et al. Multimodal Transgastric Local Pancreatic Hypothermia Reduces Severity of Acute Pancreatitis in Rats and Increases Survival. Gastroenterology 2019;156:735-747.e10.
3. Navina S, Acharya C, DeLany JP, et al. Lipotoxicity causes multisystem organ failure and exacerbates acute pancreatitis in obesity. Sci Transl Med 2011;3:107ra110.
4. Yang F, Wang Y, Sternfeld L, et al. The role of free fatty acids, pancreatic lipase and Ca+ signalling in injury of isolated acinar cells and pancreatitis model in lipoprotein lipase-deficient mice. Acta Physiol (Oxf) 2009;195:13-28.
5. Criddle DN, Murphy J, Fistetto G, et al. Fatty acid ethyl esters cause pancreatic calcium toxicity via inositol trisphosphate receptors and loss of ATP synthesis. Gastroenterology 2006;130:781-93.
6. Criddle DN, Raraty MG, Neoptolemos JP, et al. Ethanol toxicity in pancreatic acinar cells: mediation by nonoxidative fatty acid metabolites. Proc Natl Acad Sci U S A 2004;101:10738-43.
7. Bosques-Padilla FJ, Vázquez-Elizondo G, González-Santiago O, et al. Hypertriglyceridemia-induced pancreatitis and risk of persistent systemic inflammatory response syndrome. Am J Med Sci 2015;349:206-211.
8. Nawaz H, Koutroumpakis E, Easler J, et al. Elevated serum triglycerides are independently associated with persistent organ failure in acute pancreatitis. Am J Gastroenterol 2015;110:1497-1503.
9. Wan J, He W, Zhu Y, et al. Stratified analysis and clinical significance of elevated serum triglyceride levels in early acute pancreatitis: a retrospective study. Lipids Health Dis 2017;16:124.
10. Pascual I, Sanahuja A, García N, et al. Association of elevated serum triglyceride levels with a more severe course of acute pancreatitis: Cohort analysis of 1457 patients. Pancreatology 2019;19:623-629.
11. Zhang R, Deng L, Jin T, et al. Hypertriglyceridaemia-associated acute pancreatitis: diagnosis and impact on severity. HPB (Oxford) 2019;21:1240-1249.
12. Adiamah A, Psaltis E, Crook M, et al. A systematic review of the epidemiology, pathophysiology and current management of hyperlipidaemic pancreatitis. Clin Nutr 2018;37:1810-1822.
13. Crockett SD, Wani S, Gardner TB, et al. American Gastroenterological Association Institute Guideline on Initial Management of Acute Pancreatitis. Gastroenterology 2018;154:1096-1101.

14. Tenner S, Baillie J, DeWitt J, et al. American College of Gastroenterology guideline: management of acute pancreatitis. Am J Gastroenterol 2013;108:1400-15; 1416.
15. IAP/APA evidence-based guidelines for the management of acute pancreatitis. Pancreatology 2013;13:e1-15.
16. He WH, Yu M, Zhu Y, et al. Emergent Triglyceride-lowering Therapy With Early High-volume Hemofiltration Against Low-Molecular-Weight Heparin Combined With Insulin in Hypertriglyceridemic Pancreatitis: A Prospective Randomized Controlled Trial. J Clin Gastroenterol 2016;50:772-778.
17. Korn ED. Clearing factor, a heparin-activated lipoprotein lipase. I. Isolation and characterization of the enzyme from normal rat heart. J Biol Chem 1955;215:1-14.
18. Ong JM, Kirchgessner TG, Schotz MC, et al. Insulin increases the synthetic rate and messenger RNA level of lipoprotein lipase in isolated rat adipocytes. J Biol Chem 1988;263:12933-8.
19. Twilla JD, Mancell J. Hypertriglyceridemia-induced acute pancreatitis treated with insulin and heparin. Am J Health Syst Pharm 2012;69:213-6.
20. Olivecrona G. Role of lipoprotein lipase in lipid metabolism. Curr Opin Lipidol 2016;27:233-41.
21. Mousavi S, Moradi M, Khorshidahmad T, et al. Anti-Inflammatory Effects of Heparin and Its Derivatives: A Systematic Review. Adv Pharmacol Sci 2015;2015:507151.
22. Mulloy B, Hogwood J, Gray E, et al. Pharmacology of Heparin and Related Drugs. Pharmacol Rev 2016;68:76-141.
23. Schünemann HJ, Cushman M, Burnett AE, et al. American Society of Hematology 2018 guidelines for management of venous thromboembolism: prophylaxis for hospitalized and nonhospitalized medical patients. Blood Adv 2018;2:3198-3225.
24. Lu XS, Qiu F, Li JQ, et al. Low molecular weight heparin in the treatment of severe acute pancreatitis: a multiple centre prospective clinical study. Asian J Surg 2009;32:89-94.
25. Näsström B, Stegmayr B, Gupta J, et al. A single bolus of a low molecular weight heparin to patients on haemodialysis depletes lipoprotein lipase stores and retards triglyceride clearing. Nephrol Dial Transplant 2005;20:1172-9.
26. Näsström B, Olivecrona G, Olivecrona T, et al. Lipoprotein lipase during continuous heparin infusion: tissue stores become partially depleted. J Lab Clin Med 2001;138:206-13.
27. Weintraub M, Rassin T, Eisenberg S, et al. Continuous intravenous heparin administration in humans causes a decrease in serum lipolytic activity and accumulation of chylomicrons in circulation. J Lipid Res 1994;35:229-238.
28. Banks PA, Bollen TL, Dervenis C, et al. Classification of acute pancreatitis—2012: revision of the Atlanta classification and definitions by international consensus. Gut 2013;62:102-111.
29. Caprini JA. Thrombosis risk assessment as a guide to quality patient care. Dis Mon 2005;51:70-78.
30. Alagözlü H, Cindoruk M, Karakan T, et al. Heparin and insulin in the treatment of hypertriglyceridemia-induced severe acute pancreatitis. Dig Dis Sci 2006;51:931-933.
31. Singla AA, Ting F, Singla A. Acute pancreatitis secondary to diabetic ketoacidosis induced hypertriglyceridemia in a young adult with undiagnosed type 2 diabetes. JOP 2015;16:201-204.
32. McMahon MM, Nystrom E, Braunschweig C, et al. A.S.P.E.N. clinical guidelines: nutrition

support of adult patients with hyperglycemia. JPEN J Parenter Enteral Nutr 2013;37:23-36.
