# Supplementary material for: Low-Molecular-Weight Heparin Plus Insulin in Hypertriglyceridemic Acute Pancreatitis: A Randomized Clinical Trial
Source: JAMA Netw Open. 2025 Nov 7;8(11):e2542124. doi: 10.1001/jamanetworkopen.2025.42124 (PMC12595534; doi:10.1001/jamanetworkopen.2025.42124)
Supplement: Supplement 4. — Data Sharing Statement [file jamanetwopen-e2542124-s004.pdf]

Data Sharing Statement

He. Low-Molecular-Weight Heparin Plus Insulin in Hypertriglyceridemic Acute Pancreatitis. *JAMA Netw Open*. Published November 07, 2025. doi:10.1001/jamanetworkopen.2025.42124

Data
Additional Information: At the Chinese Clinical Trial Registry on June 5, 2019 (chictr.org.cn ChiCTR1900023640).
Data available: Yes
Data types: Deidentified participant data
How to access data: Researchers should contact the corresponding author (ndyfy01977@ncu.edu.cn) to request data.
When available: With publication

Supporting Documents
Document types: Informed consent form, Other (please specify)
Additional Information: Protocol
How to access documents: At the Chinese Clinical Trial Registry (chictr.org.cn ChiCTR1900023640).
When available: With publication

Additional Information
Who can access the data: Researchers whose proposed use of the data has been approved.
Types of analyses: For meta-analysis of individual participant data.
Mechanisms of data availability: After approval of a proposal
